# Current editorial challenges: Criteria for selecting articles for publication

**DOI:** 10.17179/excli2019-2076

**Published:** 2019-12-19

**Authors:** Jan Hengstler

**Affiliations:** 1 Leibniz Research Centre for Working Environment and Human Factors

## ⁯⁯⁯⁯⁯⁯

Since its foundation in 2002, Experimental and Clinical Sciences (EXCLI Journal) published more than 760 original articles and reviews, particularly in the field of cancer research (Abbastabar et al., 2018[1]; Nojadeh et al., 2018[15]; Karimian et al., 2018[9]), cell biology (Li et al., 2017[12]; Niknami et al., 2017[14]; Ahmadi et al., 2017[2]), toxicology (Randjelovic et al., 2017[16]; Hassani et al., 2018[6]; Nakhaee and Mehrpour, 2018[13]), neurosciences (Farajdokht et al., 2017[5]; Ebrahimi et al., 2017[3]), drug discovery (Li et al., 2018[11]; Khedher et al., 2017[10]) and immunology (Fahimi et al., 2018[4]; Sarvari et al., 2018[17]). However, the editors are keen to keep a broad view of science and technology and also welcome manuscripts from other fields of life sciences and interdisciplinary studies. Our main criterion during the review process is the scientific quality of the study. Editors and reviewers focus particularly on whether the methods are sufficiently described, results are presented in a transparent way, have been sufficiently reproduced in independent experiments and justify the main conclusions. If this is the case, also confirmatory studies or studies reporting negative results may be published. Each manuscript should include a statement on the aim of the study and convincingly explain why the experiments are relevant. While a high degree of novelty is welcome, it is not our most important criterion in selecting manuscripts for publication. 

### Improvement of the peer review process

Sometimes authors complain about the length of our review process. In 2019 mean time periods were 19 days from submission to decision, and 61 days from submission to publication, which may be above the average of other journals in this field of research. Nevertheless, our main challenge in future will be to improve the quality of the review process and less to enhance its speed. It is sometimes difficult to find competent reviewers; relatively often review requests have to be sent repeatedly, because the quality of a review is not sufficient. Nevertheless, with meanwhile approximately 100 articles published per year our resource of reviewers is constantly growing. It is very important for the quality of the review process that authors of EXCLI Journal accept our invitations as peer reviewers when manuscripts in their field of expertise are submitted. 

### Publish your raw data

Publication of raw data is not yet obligatory but reviewers request more frequently that the raw data should be made available in an additional electronic supplement. Raw data is currently published by an increasing fraction of articles (e.g. Hessel-Pras et al., 2018[7]; Yaripour et al., 2018[18]; Kang et al., 2018[8]). In future, the editors will even more rigorously control the quality of raw data to make sure that they are clearly assigned to the main figures and tables and sufficiently explained in legends. Typically, our reviewers asked for supplemental raw data in case of (1) animal experiments where the results of each individual animal must be presented; (2) articles where only representative results are shown in the main part. This is often the case for 'representative' Western blots. In such cases, we ask to present all blots of the independent experiments in the supplement. Usually, the full-length Western blots must be shown; (3) in case of studies with humans, authors must accept that anonymized raw data has to be published in a supplement. Of course, any information that reveals the identity of an individual will not be published. However, EXCLI Journal expects that e.g. a table with patient number, age (only years), male/female, and blood concentrations of a cytokine (or a similar piece of information) will be made available to our readers. Omics data (e.g. RNA-seq raw data) has to be made available in the established public databases. 

### Retractions and editorial strategies against scientific misconduct

Although the majority of submissions to EXCLI Journal is sound, we unfortunately increasingly receive faked manuscripts. For example, in 2018 and 2019 we received several series of more than ten manuscripts in relatively short periods of time that were formally professionally prepared, included a similar set of techniques and were presented in a similar image format. When reviewers identified suspicious data patterns, we asked all corresponding authors for raw data and despite of several initiatives never received any response. Of course, all these manuscripts were rejected and are kept on file. In a further sad case, a scientist, who acted as a reviewer for another journal, submitted the reviewed and rejected manuscript (written by other authors) to our journal (under his authorship). As soon as this case was reported, the manuscript was of course retracted. Moreover, another author copied and pasted parts of an already published review of other authors and submitted the faked manuscript to us. Since we did not yet routinely use plagiarism software in these days, the copy-paste-plagiarism was unfortunately not detected by the editors and reviewers and the review had to be rejected after publication. The editors of EXCLI Journal are sometimes surprised by the lack of foresight; even if a forged manuscript passes our controls, it will certainly be identified by readers and subsequently be retracted. 

### No fees for authors and readers

EXCLI Journal does not take any fees from authors, nor fees from readers accessing the published papers. Therefore, we have to manage the journal with fewer resources compared to commercial publishing houses and depend on the collaboration of members of the scientific community. If you wish to join the board of reviewers or editors, please do not hesitate to send your CV and list of publications for evaluation. 
